# How reliable are the effects of self-control training?: A re-examination using self-report and physical measures

**DOI:** 10.1371/journal.pone.0178814

**Published:** 2017-06-08

**Authors:** Brian M. Lee, Markus Kemmelmeier

**Affiliations:** Interdisciplinary Social Psychology Ph.D. Program, University of Nevada, Reno, Nevada, United States of America; Radboud Universiteit, NETHERLANDS

## Abstract

In light of recent challenges to the strength model of self-control, our study re-examines the effects of self-control training on established physical and self-report measures of self-control. We also examined whether beliefs about the malleability of self-control qualify any training effects. Participants in the training condition were assigned to increase use of their non-dominant hand for two weeks, and did comply mainly if they held high-malleability beliefs; yet, compared to a control condition, the physical measure of self-control did not improve. This was also evident in a secondary objective measure of self-control, a Stroop task, as well as in self-reported self-control. The discussion focuses on the lack of replication of training effects on self-control.

## Introduction

Self-control is one of the most important human endowments, as it allows people to limit impulsive behaviors [1]. Poor self-control has been found to be related to numerous problems, such as obesity, criminality, risky sexual behavior, drug and alcohol use, as well as other negative outcomes [2–6]. Conversely, high self-control has been found to be related to better grades, less psychopathology, better relationships, better interpersonal skills, healthier eating habits, better emotional control, as well as other positive outcomes [4, 7–9].

The strength model of self-control argues that self-control behaves like a muscle in that it becomes weakened from active use and people are less successful at using self-control on subsequent tasks which require its use [4]. Similarly, repeated use can strengthen self-control, much like exercising a muscle, making it less susceptible to becoming weakened from active use and leading to better subsequent outcomes on tasks which also require self-control. Thus, self-control seems to increase following training. A meta-analysis by Hagger, Wood, Stiff, and Chatzisarantis [10] reported an overall positive effect of training on improving self-control, though few studies have examined how long effects persist after the end of the training. Hui, Wright, Stewart, Simmons, Eaton, and Nolte [11] documented some persistence of effects, but findings were complicated by the specific domain of the research (dental hygiene). Muraven [12] tracked participants for a month following training, but again, the persistence of effects was domain specific (smoking cessation). Recently, Bertrams and Schmeichel [13] found that training effects were not sustained after the end of training, contradicting the previous findings.

With the persistence of self-control training (SCT) effects being unclear, the first goal of the current study was to examine the robustness of SCT effects. Such an examination is pertinent because a recent meta–analysis by Carter and McCullough [14] showed that there is a publication bias with regard to research on self-control depletion. This raises potential doubt with regard to other self-control research, especially because informal communications with self-control researchers revealed that unsuccessful and unpublished training studies exist, pointing to a file-drawer problem [15]. Moreover, a meta-analysis using alternative procedures from those by Hagger et al. [10] obtained somewhat smaller effect sizes than reported by these authors [16].

A second goal of this study was to examine the role of implicit beliefs in SCT. Research on self-control highlights the importance of implicit beliefs about self-control. Job, Dweck, and Walton [17] found that whether participants’ believed willpower to be a limited resource or not moderated depletion effects. Participants who did not believe willpower to be limited did not demonstrate reduced self-control after having engaged in a depleting task [17, 18]. Similarly, it may be possible that implicit beliefs about the trainability of self-control impact whether or not SCT effects manifest following training. Thus, we developed a scale which measures implicit beliefs about the malleability of self-control. We hypothesized that beliefs in the malleability of self-control would be linked with (a) greater compliance with the training instructions, and ultimately (b) greater success of the SCT.

Participants attended three study sessions, each two weeks apart. In the first session they were randomly assigned to an established SCT condition or a control condition and asked to follow instructions during the period between Session 1 and 2. During this time they also reported their compliance with instructions via a website. Participants did not receive any instructions for the period between Session 2 and 3. At all three sessions participants completed a set of self-report measures and completed the physical self-control task.

Notably, we used a SCT task and a physical self-control measure which are both well-established in the literature, but which are similar in nature. Our SCT asked participants to increase the use of their non-dominant hand. This procedure has been demonstrated to increase self-control, such that following this procedure participants were better able to resist the temptation to smoke [12], less likely to entertain thoughts about and engage in interpersonal aggression [19] and more resistant to self-control depletion [20]. To assess self-control, our participants were asked to squeeze a handgrip exerciser for as long as they could, with the time serving as a physical measure of self-control. The meta-analysis by Hagger et al. [10] revealed this to be a popular task when assessing self-control strength [1, 21, 22, 23]. Although muscle strength contributes to the time that a participant can hold a handgrip exerciser, previous research has documented that it is best understood as a measure of self-control as participants are required to withstand the discomfort resulting from continuing to hold the device [24, 25].

Whereas participants in the SCT condition were told to increase the use of their non-dominant hand during the training period, the physical measure was applied to both dominant and non-dominant hands. If participants use their non-dominant hand as part of the training regime, it should not be surprising if this increased use of the non-dominant hand also improves subsequent muscle strength in this particular hand. However, there is no reason to believe that such increased muscle strength in the non-dominant hand would translate to improved performance in the dominant hand *unless* the training also improved self-control. Muraven et al. [1] used participants’ ability to squeeze a handgrip as a dependent variable in assessing changes in self-control, but did not mention whether participants used their dominant or their non-dominant hand to do so [21, 23]. Note that Muraven et al.’s [1] training regime did not involve use of any particular hand; hence, these authors seemed to consider this issue unimportant enough to not even report which hand was used, nor whether participants used the same hand before and after the training. In contrast, the present investigation does make use of a SCT procedure focused on the non-dominant hand, thus making it imperative to differentiate possible effects on muscle-strength improvement from self-control improvements by assessing performance on the handgrip task with each hand in all sessions.

Additionally, a subset of participants completed a second objective measure of self-control, namely, a Stroop task. This secondary measure was included to confirm potential findings pertaining to the physical measure. The Stroop task, in which participants are shown color words in various font colors and indicate the color of the word while ignoring what the word actually says, has also been used in previous self-control research, and is another popular measure for self-control [10, 26, 27].

## Method

### Participants

All participants provided written informed consent at the initial session, prior to engaging in the research activities. The consent forms and procedures were approved by the University of Nevada, Reno Social Behavior and Education IRB (approval number 508754–5). Across four different semesters, 147 students (74.8% female; 67.6% White) participated in the study in exchange for course credit and $15 (USD). The study consisted of three lab sessions.

Assuming the estimate for training effects reported by Hagger et al. [10], *d* = 1.07 (p. 510, averaged corrected standardized difference effect size), and assuming α = .05 and β = .99, the required sample size was 68 to detect an effect of this magnitude. This estimate pertains to the comparison between a training condition and a control condition following the training. Only after the study was completed and the present manuscript submitted for publication did the authors learn about two recent meta-analyses that estimated the effects to be smaller, Hedges’ *g* = .30 [28], and Hedges’ *g* = .36 [29] (note that Hedges’ *g* and Cohen’s *d* only differ in that *g* includes a correction factor for sample size).

One can question whether a simple comparison between a control group and a self-control training group does provide the correct basis for a sample size estimate because we employed a mixed-model design which was predicated on the notion that the control group and the training group would diverge over time. Indeed, ours was a mixed-model design, in which we assigned each participant to an experimental condition (between-groups factor) but assessed each participant in three different sessions (repeated-measures factor) on both their dominant and nondominant hand (repeated-measures factor), for which we expected differential effects over time. Hence, it was important to determine the sensitivity of this design for a Condition x Session x Hand interaction. Assuming α = .05 and β = .80, as well as an average correlation of *r* = .60 between measurements, our planned sample size of 68 was able to detect a Condition x Hand x Session interaction effect with an effect size that corresponded to a Cohen’s *d* of .23—arguably a small effect size. Note that, other than the estimates reported by previous authors [10, 28, 29] we do not focus on a simple comparison between a training group and a control group post-training. Rather, our estimate pertains to the differential change that emerges between these two groups over the course of the training.

The data were collected in two batches. In the first set of data, 108 students participated across three successive semesters. After removal of partial completers and ambidextrous students, the number of usable cases reached 65, thus making it necessary to collect additional data. Because preliminary analyses of the incomplete data set revealed ambiguous results, in the final semester of data collection (resulting in 39 more participants), we added one dependent variable to the very end of the procedure to examine the consistency of potential findings across measures.

### Procedure

#### Session 1

At the first session, participants provided written consent, and then completed all of the self-control measures (discussed below). Participants were given the self-report measures first, then the physical measure, and lastly, for the second batch, the Stroop task. Participants were randomly assigned to conditions via a coin toss and received corresponding instructions.

#### SCT condition

Participants in the training condition were instructed to use their non-dominant hand for mundane, daily tasks (e.g., carrying objects, brushing their teeth, stirring beverages) between the hours of 8AM and 6PM. This training program is modeled after the exercises in Gailliot et al. [20] (see Study 2 & 4); also [19], which was previously demonstrated to bolster self-control.

#### Control condition

Participants assigned to the control condition were instructed to keep a journal of the temptations they encountered daily (e.g., not doing homework when they ought to, making poor eating choices, hanging out with friends instead of studying) between the hours of 8AM and 6PM, and to do so whether they resisted the temptation or capitulated. This control condition was modeled after Muraven [12], and served the purpose of making self-control behaviors salient, without requiring participants to practice self-control. Notably, keeping a journal has been occasionally used in the context of a self-control manipulation [1, 8, 9, 30]. However, diaries were either used to record participants’ activities [1, 8, 9, 30], or to record participants’ food intake [1]. Even though in Muraven et al. [1] there were no explicit instructions to modify food intake while keeping a food diary, keeping a food journal may lead to a spontaneous modification of behavior, though effects are small [31, 32]. Nevertheless, the task of recording one’s food intake might be reactive and increase the likelihood of people exercising self-control. However, a similar process is much less likely to occur for the recording of daily temptations, which are experienced often as spontaneous and outside of the individual’s volition. Consistent with this notion, Hufford et al. [33] found little evidence for problem drinkers tasked with recording their alcohol-related temptations to alter their behavior. Moreover, Muraven [12] demonstrated that the journaling task was no different in its consequences on self-control improvement from an alternative control condition (performing 3–5 minutes of simple math problems twice a day).

Participants in both conditions were told to follow instructions for a period of two weeks ending with Session 2.

#### Interim between Session 1 and Session 2

All participants regardless of condition received text messages every three days reminding them to follow condition-specific study instructions. Participants were also reminded to complete an online survey which inquired about their levels of compliance with instructions. For participants in the SCT condition the survey asked how often participants engaged in using their non-dominant hand, and for participants in the control condition how often they recorded temptations in their journal. Ratings were made on a 7-point scale (1 *Not At All*, 7 *Very Often*). Each time they accessed the online survey, participants listed examples of their use of their non-dominant hand or the temptations they encountered.

#### Session 2

Two weeks after Session 1, participants returned to the lab to complete all measures previously assessed in Session 1. Moreover, at the end of Session 2, participants were instructed to no longer engage in the condition-specific instructions. Additionally, no further text message reminders were sent to participants between Session 2 and Session 3.

#### Session 3

Again, participants completed all measures from Session 1 and Session 2. At the end of Session 3, participants were debriefed.

### Measures

Across three sessions, participants received the same set of self-report measures, with participants indicating how much an item applied to them or how much they agreed with it (1 *Not At All*, 7 *Very Much*).

#### Self-reported self-control

Levels of self-control were assessed using the 13-item Brief Self-Control Scale [7] (*α* = .81, .85 and .84 for Sessions 1, 2, and 3, respectively) as well as the 24-item Low Self-Control Scale [34], a popular scale in criminology with reliabilities (α = .82, .87, and .89). Scales were scored such that higher values always indicated greater levels of self-control. The authors of these scales generally conceive of one’s capacity to exercise self-control as a disposition [7, 34, 35]. However, with training being able to induce changes in self-control over time [1, 8, 9, 12, 20, 30], it should not be surprising that authors have frequently reported changes in self-reported self-control over time [36–38].

#### Implicit beliefs about depletability of self-control

Participants completed the scale by Job et al. [17], which assessed whether participants believed that self-control was a limited resource and would be depleted whenever participants engaged in the exercise of self-control. We used the 12-item version employed in their Study 4, which included both subscales pertaining to the effects of strenuous mental activity as well as the effects of resisting temptation (*α* = .83, .88 and .89). (Results did not change if we only employed the subset of six items, which represented the scale used by Job et al. [17] in their Study 1.) Higher scores indicate a greater belief in self-control being a *non*-depletable resource.

#### Implicit beliefs about malleability of self-control

**Fig 1:**
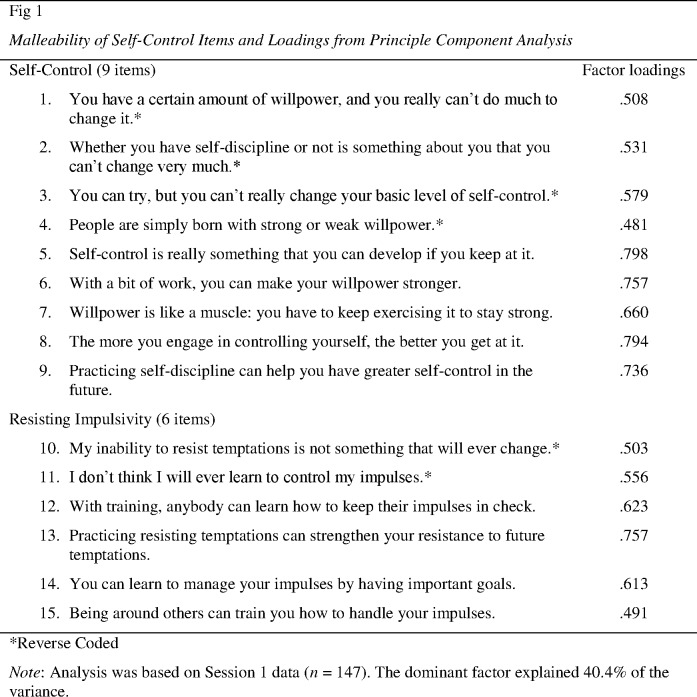
Malleability of self-control items and loadings from principle component analysis. Analysis was based on Session 1 data (*n* = 147). The dominant factor explained 40.4% of the variance.

We generated a novel scale that measured participants’ beliefs as to whether their capacity to exercise self-control is malleable, and whether their capacity to resist temptation is malleable. Parallel to Job et al. [17], we generated 10 items addressing malleability of self-control and 10 items pertaining to resisting impulsivity. Based on principal component analyses, we reduced the number of items in the malleability of self-control scale to nine, and the number of items of the resisting impulsivity scale to six (see Fig 1 for final items). Reliabilities of the two scales were satisfactory, all *α* > .73, across the three points in time. Because all items loaded on the same factor, focusing on Session 1 data we performed a confirmatory factor analysis to find that a two-factor solution, distinguishing malleability of self-control and resisting impulsivity, reveal a slightly better model fit than a single-factor solution, AIC = 7269.63 vs. 7272.75, -2 *LL* = 7177.64 vs. 7182.76, likelihood ratio test χ^2^(1) = 5.12, *p* = .024. The same pattern was obtained for Session 2 and 3 data. However, the subscales were highly correlated (*r* = .68, .66 and .78), and for results presented here they were combined into a single scale, *α* = .87, .90 and .91 (Sessions 1, 2 and 3, respectively), as subsequent analyses did not reveal any discernable difference in findings using a single scale or two parallel scales.

#### Physical measure of self-control

Following completion of the self-report measures, participants were given the handgrip task previously employed by other researchers [1, 21, 23]. Specifically, participants were asked to hold a handgrip exerciser closed as long as they could, which was timed. To determine when the participant’s hand was no longer applying the necessary amount of force to maintain closure of the handgrip exerciser, a small foam square was inserted between the handles. When the foam square fell out, the timer was stopped. The length of time that participants were able to squeeze the handgrip exerciser served as a physical measure of self-control [1, 21, 23]. Other than previous authors who did not report which hand participants used to squeeze the handgrip exerciser [1, 21, 23], we recorded participants’ times separately for both their dominant hand and their non-dominant hand.

#### Stroop

After completing the handgrip exerciser task a subset of participants (*n* = 39), all of whom participated in the fourth of the four semesters during which this study was run, were asked to complete a color-word Stroop task (available through millisecond.com) in English. Participants were shown words (“red”, “blue”, “green”, or “black”) which were presented in various font colors. In some instances, the word and its font color were congruent. In others, they were incongruent. Participants were to indicate the color of the font while ignoring what the word actually said. Also, solid blocks of color were displayed, which served as a control. Participants’ response times were recorded in milliseconds, which served as a secondary physical measure of self-control. A total of 84 trials are given to each participant (4 colors x 3 color-stimulus congruency (congruent, incongruent, control) x 7 repetitions).

## Results

### Participant signup, compliance and retention

#### Time of signup

**Fig 2:**
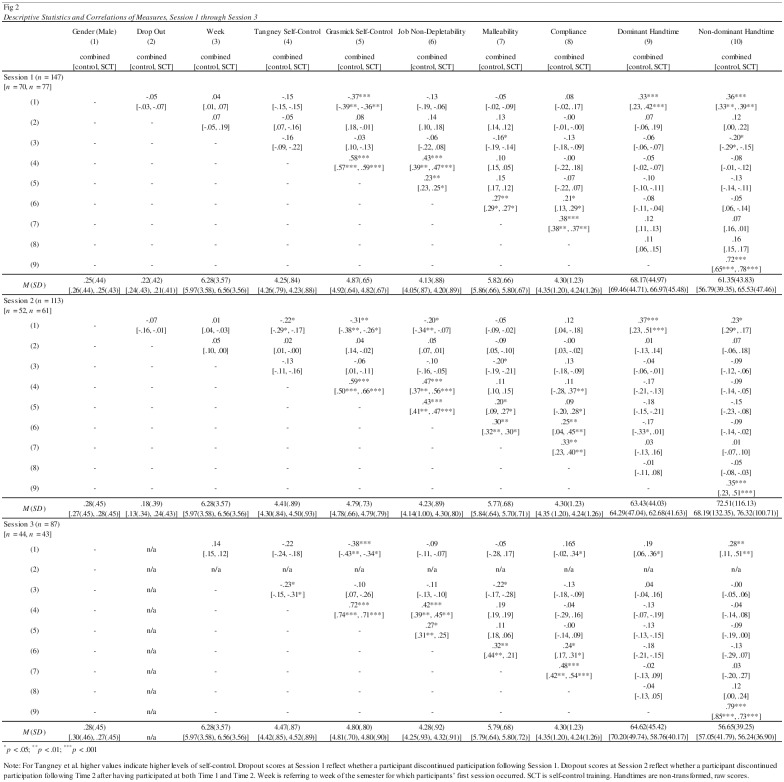
Descriptive statistics and correlations of measures, Session 1 through Session 3. For Tangney et al. higher values indicate higher levels of self-control. Dropout scores at Session 1 reflect whether a participant discontinued participation following Session 1. Dropout scores at Session 2 reflect whether a participant discontinued participation following Time 2 after having participated at both Time 1 and Time 2. Week is referring to week of the semester for which participants’ first session occurred. SCT is self-control training. Handtimes are non-transformed, raw scores.

Participants signed up for the study anytime between the first and the twelfth week of a 16-week semester. Because higher levels of self-control in participants might be linked to completing course-related responsibilities sooner rather than later, we examined whether individual differences related to self-control, malleability beliefs and non-depletability beliefs predicted when in the semester participants joined the study [39, 40]. Zero-order correlations suggested that people who believed that self-control was malleable (as measured at Session 1) were more likely to sign up earlier in the semester (see Fig 2). When week of the semester of participants’ first session of the study was regressed onto these three predictors (assessed at Session 1, using Tangney et al. [7] as the measure of self-control) there was only a trend for individuals with higher malleability beliefs to sign up earlier in the semester, *b* = -0.78, *se* = 0.46, *p* = .093 (controlling gender and age). When this model was run again using Grasmick et al. [34] as the measure of self-control, very similar results emerged. No predictors were significant, and again, there was only a trend for individuals with higher malleability beliefs to sign up earlier in the semester, *b* = -0.76, *se* = 0.46, *p* = .105. This is consistent with the notion that believing in the malleability of self-control was part of the motivation of signing up for the study early in the term, as the study was advertised as a self-control training study. Importantly, a separate analysis of variance showed that there was no difference in the week of the semester during which participants in the training condition and the control condition participated in the study, *F* < 1.

#### Retention

There was considerable loss of research participants over the course of the study. Whereas 147 students participated in Session 1, 113 attended Session 2, and 87 attended Session 3. Although a substantial decline, our retention rate was comparable to other SCT studies using student samples [11, 41, 42], though other than in [41], we did not observe any differential attrition by experimental condition. However, most studies using student samples do not report any information concerning participants terminating a multi-week study prematurely [1, 12, 19, 43]. Because dropout might reflect lack of self-control, we predicted retention using logistic regression based on trait self-control (as before, one model using Tangney et al. [7] and another model using Grasmick et al. [34]), malleability beliefs (at Session 1), non-depletability beliefs (at Session 1), age, and gender. No effects emerged. Dropout between Session 2 and Session 3 was also examined with logistic regression using the same predictors, and again, no effects emerged.

#### Compliance

We computed average compliance ratings (*M* = 4.30, *SD* = 1.23; range 1.33–7.00) based on all ratings that participants provided between Session 1 and Session 2, regardless of how many such ratings participants had provided. Participants were expected to submit four compliance ratings (median = 3), though a total of 16.3% of participants did not submit any compliance ratings, whereas 11.6% participants reported more than the expected number of four (maximum of 6). Notably, the SCT and control conditions did not differ with regard to the average compliance ratings nor in the number of such ratings that were received, both *F* < 1. Compliance did not change over time either, meaning that participants’ first, second, third and fourth self-reported compliance scores were compared, *F*(3, 256.5) = 0.36, *p* = .78.

Malleability beliefs at Session 1 correlated with the average compliance ratings during the subsequent two weeks, though non-depletability beliefs showed a similar correlation (see Fig 2). An analysis in which average compliance was regressed on Session 1 malleability beliefs, non-depletability beliefs, self-reported self-control (Tangney et al. [7] measure), age, and gender showed that malleability beliefs, *b* = .61, *se* = .16, *p* < .001, as well as non-depletability beliefs, *b* = .26, *se* = .13, *p* = .050, predicted compliance. The same model using Grasmick et al. [34] self-reported self-control revealed very similar results. Malleability beliefs predicted compliance, *b* = .62, *se* = .16, *p* < .001, and non-depletability beliefs was nearing significance as well, *b* = .24, *se* = .12, *p* = .054.

### Self-report measures: Stability over time and correlations

We report analyses including participants who contributed to all three sessions of the study; though we also conducted comparisons including all participants who contributed to Session 1 and Session 2. Results were consistent. Fig 2 provides means and standard deviations for all measures, displayed for each point in time in the bottom row of each panel. It also shows those for each experimental condition separately for additional information.

We used a series of general linear models to examine changes over time in each variable by condition over time. Means for Job et al.’s non-depletability measure [17] increased over time, *F*(2, 82) = 7.95, *p* = .001, η_p_^2^ = .16. Examination of simple slopes showed that there was a substantive change between Session 1 and Session 2, *p* = .003, and between Session 1 and Session 3, *p* < .001, even when there was no change between Session 2 and Session 3, *p* = .10. Malleability means did not vary over time, *F* < 1. However, there was an increase in self-reported self-control with the Tangney measure, *F*(2, 83) = 10.04, *p* < .001, η_p_^2^ = .20, with scores increasing between Session 1 and Session 2, *p* = .001, and Session 1 and Session 3, *p* < .001, but no change between Session 2 and Session 3, *p* = .080. No change over time occurred with the Grasmick measure of self-reported self-control though, *F*(2, 83) = 0.77, *p* = .47, η_p_^2^ = .02. Across sessions, all three scores were highly correlated, non-depletability all *r* > .75, *p* < .001, malleability beliefs all *r* > .79, *p* < .001, Tangney self-reported self-control all *r* > .84, *p* < .001, and Grasmick self-reported self-control all *r* > .83, *p* < .001. Fig 2 also displays the correlations of measures with one another at all three sessions. The pattern was remarkably consistent over time, but experimental condition never produced any significant effect. Notably, Job et al.’s [17] non-depletability beliefs were related to both measures of self-reported self-control [7, 34].

Of particular interest were the correlations pertaining to the new measures of malleability beliefs. This scale did not correlate with self-reported self-control. There was, however, an association between malleability beliefs and non-depletability beliefs, which emerged at all three points in time. This pattern is consistent with the notion that malleability beliefs are not redundant with trait self-control, and that they are conceptually distinct from, even though empirically related to, non-depletability beliefs.

### Effects of SCT

#### Physical self-control

Times of how long participants squeezed the handgrip exerciser with their dominant and non-dominant hand at the three different sessions were analyzed using a multilevel analysis. Specifically, because of the non-independent data structure, we used a generalized linear mixed model that took account of the fact that both times for the dominant and non-dominant hands across three sessions were nested within the same participant. This model allowed for performance comparisons between the two hands and, more importantly, an analysis of differential changes in the performance of each hand over time. The error covariance matrix assumed that errors at different points in time were independent from one another. We expected to observe a differential change for the dominant and non-dominant hand over time for participants in the control and the training condition. Our initial generalized linear mixed model included Hand (dominant vs. non-dominant), Session (1–3), Condition (SCT vs. control) as categorical predictors. Condition was modeled as a between-participant fixed effect, whereas Hand and Session were modeled as within-participant random effects. This analysis was based on those participants who had contributed data in all three sessions. One ambidextrous person was excluded because it was not possible to distinguish a dominant and a non-dominant hand, leaving 79 participants—a number greater than the sample size requirement discussed earlier. Prior to analysis, all hand-times were submitted to a log-transformation to normalize distributions.

This model (-2 *LL* = 666.92, *AIC* = 685.32) revealed a main effect for Hand showing individuals had longer hand-times for their dominant hand than their non-dominant hand (*M*_*log(s)*_ = 3.94 vs. 3.81), *F*(1, 52) = 7.60, *p* = .008, which was qualified by interactions with Session, *F*(2, 29) = 3.96, *p* = .03, and condition, *F*(1, 52) = 4.93, *p* = .031. However, the anticipated three-way interaction showing a training effect on the dominant hand nor any other effect did not approach significance, *F*s < 1.19, *p*s > .31. With 79 participants and assuming α = .05, β = .80, and with an observed average *r* = .62, this analysis should have been able to detect a small interaction effect of *d* = .21.

As discussed earlier, we were interested if implicit beliefs about the malleability of self-control would moderate such changes over time. Therefore, we added to our model malleability beliefs (at Session 1) and average compliance ratings as continuous predictors to test for possible moderator effects. All interactions involving the three factors critical to our experimental design, Hand, Session, Condition, and either malleability beliefs or average compliance were included. That is, we generated all possible interactions, but never allowed malleability beliefs and average compliance to interact with each other. To control for other extraneous effects, we also controlled for gender, week of sign up, and depletability beliefs at Session 1 (all main effects only). The resulting model fit the data worse than the above simpler model (-2 *LL* = 685.42, *AIC* = 703.88). In addition to the Hand main effect, *F*(1,8) = 8.65, *p* = .018, the Hand by Session interaction, *F*(2,8) = 4.97, *p* = .039, and the Hand by Condition interaction, *F*(1,8) = 6.83, *p* = .033, we only found a tendency for men to squeeze the handgrip exerciser longer than women, *M*_*log(s)*_ = 3.63 vs. 4.21), *F*(1, 2) = 14.92, *p* = .071. No other significant effects were found, all *p*s > .15.

Because multilevel models are based on maximum likelihood estimation, they are quite tolerant for missing values, which allowed us to relax inclusion criteria and add participants’ data into the analysis who had completed two of the three sessions in addition to those that had completed all three sessions (total *n* = 90). Despite this larger sample size and increased statistical power, this analysis produced identical findings to the model above.

The above analyses were also run as a three-way model in which hand times were treated as nested within each of the three sessions, which in turn were all nested within the same participants. This model yielded essentially the same result, except that the above gender effect emerged more strongly, *F*(1, 9) = 15.37, *p* = .003, and that it also revealed an effect for week of the semester of participants’ first session, *F*(11, 401) = 3.971, *p* < .001. Thus, the present findings do not support that training one’s non-dominant hand produces increased performance in either hand, i.e., increased performance in self-control.

#### Self-report measure of self-control

Self-report measures of self-control were entered into a two-level mixed model to account for three consecutive self-reports of self-control being nested within participants. We were interested in any differential change between the control and SCT conditions, whether malleability beliefs would moderate such changes over time, and if compliance with the instructions qualified these changes. The mixed model used the Tangney et al. [7] self-reported self-control scale as the dependent variable, Session (1–3), and Condition (training vs. control) as factors, and malleability beliefs and compliance as continuous predictors. Gender, week of semester of participants’ first session, and depletability beliefs were added as main effects only to control for their potential effects. Analyses were based only on those participants who had contributed data at all three sessions, leaving 82 participants. Depletability beliefs as a main effect approached significance, *F*(1, 63.00) = 2.62, *p* = .080; however, no other effects emerged, all *p*s > .31. The same mixed model using the Grasmick et al. [34] measure as the dependent variable yielded no significant effects, all *p*s > .26. The present findings do not support that training self-control through increased use of the non-dominant hand leads to increased self-reported self-control.

#### Stroop

A subset of participants (*n* = 38) had taken a Stroop task as a secondary objective measure of self-control. Similar to the other tasks, because of participant attrition, only 20 of these participants had completed all three sessions. One participant had extreme scores. Another participant was not a native English speaker, thus making the Stroop task (which was performed in English) difficult to interpret for that participant. These two participants were removed from subsequent analyses for these reasons. For participants for whom there were two sessions completed of the three, multiple imputation through use of Stata [44] was utilized to generate data for the missing data points for Session 3. The resultant number of participants for the subsequent analyses was 23.

We calculated Stroop scores by subtracting congruent times from incongruent times, as well as calculating an adjusted Stroop score by dividing the above Stroop score by control times to adjust for overall speed of responses. Both Stroop scores as well as adjusted Stroop scores were entered into two-level mixed models to account for three consecutive Stroop tasks being nested within participants. As in the previous models, we were interested in any differential change between experimental conditions, whether malleability beliefs would moderate such changes over time, and if compliance with the instructions qualified these changes. The mixed model included the Stroop scores and then the adjusted Stroop scores as the dependent variable, Session (1–3) and Condition (training vs. control) as factors, and malleability beliefs and compliance as continuous predictors. Gender, week of participation, and depletability beliefs were controlled for (i.e., added as main effects only). No significant effects or interactions emerged with either model, all *p*s > .086. The findings do not support that training with the non-dominant hand leads to improved Stroop task performance. However, this result must be interpreted with caution because only a subgroup of our participants completed the Stroop task. This particular aspect adds to the present study’s pattern of non-supportive findings, albeit remains inconclusive, as it is likely underpowered.

## Discussion

The present investigation yielded only null findings. On the one hand, our study provided behavioral evidence that participants in the SCT condition adhered to experimental instructions. This was evident in self-reported compliance, which has been used in previous research [45], as well as the act of reporting their compliance online, i.e., number of compliance checks completed. This finding is surprising in light of much of the published evidence and appears consistent with the criticism leveraged against research on the effects of self-control training, which has been suspected to be compromised [14, 16].

Similarly, our attempt to examine whether subjective beliefs about the malleability of one’s self-control capacity moderate the expected effects ultimately failed. Although our novel scale revealed good psychometric qualities and showed satisfactory convergent and divergent validity, malleability scores did not moderate training effects, as the latter did not materialize.

A possible criticism of the present study is that the SCT instructions might have drawn attention to the non-dominant hand, thus signaling to participants that experimenters were interested in the non-dominant hand, but not the dominant hand. However, there was no change in the performance of the dominant hand that would support the claim, there were no changes in Stroop performance, nor were there changes in self-reported self-control. Overall, the present research found little evidence that self-control training successfully improved individuals’ physical measures of self-control or self-reported self-control. Whereas our findings cannot refute that improving self-control is possible, the underlying processes are likely complex and difficult to translate into a simple training procedure.

### Our study in the light of recent developments

Only after completion of the present research did we became aware of two recent meta-analyses, which were unpublished at the time of submission [28, 29]. Both analyses reported small but positive effects of training on self-control. Whereas this overlap is unfortunate, the inclusion of our study would have further reduced their already low effect size estimates. Conversely, the fact that we looked toward the large effect size estimate published by Hagger et al. [10] for sample size requirements, yet to detect the type of effects emerging from the recent meta-analyses [28, 29], a larger sample size would have been required had our observed average correlation between measurements been smaller. We believe that Hagger et al. [10] only synthesized the evidence available to them and cannot be blamed for their inflated estimate (but see [16]); however, this instance may serve as evidence that publication bias does have real consequences and can mislead members of the scientific community.
